# Electroconvulsive Therapy Modulates Resting-State EEG Oscillatory Pattern and Phase Synchronization in Nodes of the Default Mode Network in Patients With Depressive Disorder

**DOI:** 10.3389/fnhum.2019.00001

**Published:** 2019-02-01

**Authors:** Akihiro Takamiya, Jinichi Hirano, Bun Yamagata, Shigeki Takei, Taishiro Kishimoto, Masaru Mimura

**Affiliations:** ^1^Department of Neuropsychiatry, School of Medicine, Keio University, Tokyo, Japan; ^2^Center for Psychiatry and Behavioral Science, Komagino Hospital, Tokyo, Japan; ^3^Department of Laboratory Medicine, School of Medicine, Keio University, Tokyo, Japan

**Keywords:** electroconvulsive therapy, electroencephalography, depressive disorder, low resolution electromagnetic tomography, current source density, phase synchronization

## Abstract

**Introduction**: Electroconvulsive therapy (ECT) has antidepressant effects, but it also has possible cognitive side effects. The effects of ECT on neuronal oscillatory pattern and phase synchronization, and the relationship between clinical response or cognitive change and electroencephalogram (EEG) measurements remain elusive.

**Methods**: Individuals with unipolar depressive disorder receiving bilateral ECT were recruited. Five minutes of resting, eyes-closed, 19-lead EEG recordings were obtained before and after a course of ECT. Non-overlapping 60 artifact-free epocs of 2-s duration were used for the analyses. We used exact low resolution electromagnetic tomography (eLORETA) to compute the whole-brain three-dimensional intracortical distribution of current source density (CSD) and phase synchronization among 28 regions-of-interest (ROIs). Paired *t*-tests were used to identify cortical voxels and connectivities showing changes after ECT. Montgomery Asberg Depression Rating Scale (MADRS) and Mini-Mental State Examination (MMSE) were used to evaluate the severity of depression and the global cognitive function. Correlation analyses were conducted to identify the relationship between changes in the EEG measurements and changes in MADRS or MMSE.

**Results**: Thirteen depressed patients (five females, mean age: 58.4 years old) were included. ECT increased theta CSD in the anterior cingulate cortex (ACC), and decreased beta CSD in the frontal pole (FP), and gamma CSD in the inferior parietal lobule (IPL). ECT increased theta phase synchronization between the posterior cingulate cortex (PCC) and the anterior frontal cortex, and decreased beta phase synchronization between the PCC and temporal regions. A decline in beta synchronization in the left hemisphere was associated with cognitive changes after ECT.

**Conclusion**: ECT modulated resting-state EEG oscillatory patterns and phase synchronization in central nodes of the default mode network (DMN). Changes in beta synchronization in the left hemisphere might explain the ECT-related cognitive side effects.

## Introduction

Electroconvulsive therapy (ECT) is the most effective antidepressant treatment (Kellner et al., 2012), but it also has potential cognitive side effects (Semkovska and McLoughlin, 2010). A better understanding of the biological mechanisms behind ECT-related antidepressant effects and cognitive side effects may have implications for developing new antidepressant treatments that have comparable efficacy to ECT without the cognitive side effects.

The electroencephalogram (EEG) is one of the principal methods for extracting information from the human brain noninvasively (Fingelkurts and Fingelkurts, 2015). Studies investigating the effects of ECT on electrophysiological measurements date back to the 1930s. Although the results of early studies using qualitative ratings were not consistent, quantitative analyses of EEG data have reported ECT-induced slow-wave increases in the fronto-temporal regions (Sackeim et al., 1996). Recent studies found that ECT-induced theta changes in the subgenual anterior cingulate cortex (ACC) were associated with improvement in psychotic symptoms (McCormick et al., 2009), and ECT modulated multi-scale entropy in depressed patients (Farzan et al., 2017). However, the number of studies examining the electrophysiological effects of ECT is still small compared to other modalities, such as magnetic resonance imaging (MRI; Abbott et al., 2014). Moreover, the relationship between changes in clinical response and/or cognitive function and changes in EEG measurements remains elusive.

Depression is now conceptualized as a system-level disorder (Mayberg et al., 2005), and it has been reported that depression showed increased resting-state EEG functional connectivity among multiple brain regions (Fingelkurts et al., 2007; Leuchter et al., 2012). The effects of antidepressant medications and transcranial magnetic stimulation (TMS) on brain electrophysiological measures have been examined by using a newly developed measurement of EEG functional connectivity, namely lagged non-linear connectivity or lagged phase synchronization (Pascual-Marqui et al., 2011; Olbrich et al., 2014; Iseger et al., 2017; Kito et al., 2017). Because phase synchronization is considered to be a fundamental neural mechanism relating to neural plasticity and cognitive processes (Fell and Axmacher, 2011), this measurement seems to be ideal for investigating the underlying mechanisms of ECT.

The aim of this study was to investigate the effects of ECT on cortical oscillatory activity and EEG phase synchronization throughout the brain. We also investigated whether changes in these EEG measurements were associated with clinical response as well as cognitive change.

## Materials and Methods

### Trial Setting

We performed a longitudinal study to compare changes in neuronal oscillatory pattern and phase synchronization before [time point (TP1): time between admission and the first ECT] vs. after ECT (TP2: within 1 week of the completion of the ECT series). This study was conducted at Keio University Hospital from June 2013 through December 2015. Ethical approval was obtained from the Ethics Committee of Keio University School of Medicine, and the study was conducted in accordance with the principles expressed in the Declaration of Helsinki. Written informed consent was obtained from all the participants.

### Participants

Individuals meeting the following inclusion criteria were recruited from Keio University Hospital: (1) International Classification of Disease 10th edition (ICD-10) diagnosis of depressive disorder (F32, F33; World Health organization, 1994); (2) inpatients at the psychiatric ward; (3) clinical indications for ECT including treatment resistance and a need for a rapid and definitive response; and (4) age ≥20 years. Exclusion criteria were the following: (1) a lifetime history of neurological or degenerative disorder; (2) unstable or severe medical illness; (3) ECT treatment within the last 3 months; (4) lifetime history of drug or alcohol misuse;and (5) difficulty in communication. These participants were originally collected for a previous study (Hirano et al., 2017).

### Clinical Assessments

The following clinical assessments were performed by trained psychiatrists who were blinded to the EEG data at TP1 and TP2. Montgomery Asberg Depression Rating Scale (MADRS; Montgomery and Asberg, 1979) was used to evaluate the severity of depression, and Mini-Mental State Examination (MMSE; Folstein et al., 1975) was used for the assessment of global cognitive function. We also collected participants’ demographic and clinical information including age, sex, past medical history, medications prescribed, and ECT data (e.g., the number of ECT sessions). Clinical response was defined as a decrease in MADRS score of at least 50% from baseline (Rush et al., 2003), and remission was defined as a total MADRS score of 10 or less (Zimmerman et al., 2004).

### ECT Treatment

ECT was performed with bitemporal electrode placement using a brief-pulse square-wave device (Thymatron system IV device; Somatics, Inc., Lake Bluff, IL, USA). The intensity of the first ECT session was determined based on the half age method. Treatments were performed three times a week, and treatments were continued until a plateau was reached and no more improvement was seen in the last two sessions. EEG seizure manifestations were monitored to ensure adequate seizure. When the EEG seizure duration was less than 25 s, the patients were restimulated at a higher intensity after a 1 min interval. Thiopental (3.5 mg/kg) was used for general anesthesia, and succinylcholine (1 mg/kg) was used to induce muscle relaxation (A Task Force Report of the American Psychiatric Association, 2001).

### EEG Recording

The participants underwent EEG before (TP1) and after (TP2) a series of ECT. The first recording was performed between admission and the first ECT, and the second recording was done within 1 week after the last ECT. EEG data was obtained and digitalized on Nihon Kohden EEG machines (Neurofax EEG-1200) by trained technicians at Keio University Hospital. Five minutes of resting EEG was recorded under eyes-closed conditions from 19 scalp locations according to the international 10/20 system (Fp1/2, F3/4, C3/4, P3/4, O1/2, F7/8, T3/4, T5/6, Fz, Cz, Pz) referenced to linked ear lobes (A1 and A2). Impedances were kept below 5 kΩ. Data were collected digitally with a sampling rate of 500 Hz. Simultaneous video recordings were used to check each segment for movements and to exclude these segments.

### EEG Preprocessing

EEG raw data was first analyzed using the EEGLAB (Delorme and Makeig, 2004). The data were downsampled to 250 Hz to reduce computing time, filtered at 1.0 Hz (high-pass) and 50 Hz (notch-filter), and segmented in 2-s epocs. Then the EEG signal was decomposed into independent components (ICs) by Infomax IC analysis (Bell and Sejnowski, 1995), using the EEGLAB *runica* command. Each IC was visually examined and ICs corresponding to artifactual sources were removed. The cleaned EEG signal was reconstructed by retro-projecting only the ICs containing a cerebral signal. The reconstructed signals were referenced to Cz and the first 60 epocs were entered into the following analyses.

### EEG-Source Localization Analysis

We used exact low resolution electromagnetic tomography (eLORETA) to compute the three-dimensional (3D) intracortical distribution of electric neuronal activity for the following six bands: delta (1.0–3.5 Hz), theta (4.0–7.5 Hz), alpha (8.0–12.0 Hz), beta 1 (12.5–20.0 Hz), beta 2 (20.5–30.0 Hz), gamma (30.5–45.0 Hz). The eLORETA method is a discrete, 3D-distributed, linear, weighted minimum norm inverse solution. Compared with previous versions, eLORETA has no localization bias in the presence of structured noise in simulated data (Pascual-Marqui, 2007a). Numerous studies using functional MRI (fMRI; Vitacco et al., 2002; Mulert et al., 2004), structural MRI (Worrell et al., 2000), positron emission tomography (PET; Pizzagalli et al., 2003; Zumsteg et al., 2005), and intracranial EEG (Zumsteg et al., 2006a,b) have validated LORETA to study brain activity. Studies using a relatively small number of electrodes (i.e., 19 electrodes) have applied LORETA source localization successfully (McCormick et al., 2009; Thatcher et al., 2014).

Several previous studies have reported abnormal current source density (CSD; Pizzagalli et al., 2002, 2004) and EEG functional connectivity (Olbrich et al., 2014) in depressed patients, as well as changes in EEG functional connectivity with antidepressant treatments, including antidepressant medications (Olbrich et al., 2014; Iseger et al., 2017), and TMS (Kito et al., 2017). The eLORETA solution space (6,239 voxels; spatial resolution; 5 mm) is restricted to the cortical gray matter. The Montreal Neurologic Institute average MRI brain (MNI152; Mazziotta et al., 2001) is used as a realistic head model for which the lead field was computed (Fuchs et al., 2002). At each voxel, LORETA values represent the power of the computed intracortical current density distribution for each frequency band. To eliminate variability for the total power changes of each subject, we used subject-wise data normalization implemented in LORETA before statistical analyses.

### EEG Functional Connectivity Analysis

We selected 28 regions-of-interest (ROIs) covering the whole-brain based on Brodmann Areas (BAs) provided in the eLORETA software, as others did in a previous study (Di Lorenzo et al., 2015; Supplementary Table S1). We selected a single voxel in the center of each ROI as the representative voxel. We used lagged phase synchronization (Kito et al., 2017) as a measure of EEG functional connectivity between all pairs of ROIs. Lagged phase synchronization quantifies the non-linear relationship between two ROIs after the instantaneous zero-lag contribution has been excluded. This correction is important because zero-lag synchronization is usually due to non-physiological artifacts, such as volume conduction and low spatial resolution that usually affect other connectivity indices (Nolte et al., 2004; Stam et al., 2007). Details on the lagged phase synchronization algorithm can be found in several reports (Pascual-Marqui, 2007b; Kito et al., 2017).

### Statistical Analysis

We conducted paired *t*-tests to compare differences in CSD and lagged phase synchronization between TP1 and TP2. We used statistical nonparametric mapping (SnPM; Nichols and Holmes, 2002). This method determined the critical probability threshold values for the actually observed *t*-values with correction for multiple comparisons across all voxels and all frequencies. A total of 5,000 permutations were conducted to calculate the critical threshold t_crit_ for *p* = 0.05 with correction for multiple comparisons among all voxels and frequencies. The omnibus null hypothesis was rejected if at least one *t*-value (i.e., voxel tmax) was above the t_crit_. The use of SnPM in eLORETA has been validated in several studies (Pascual-Marqui et al., 1999; Canuet et al., 2012). To investigate associations between EEG changes and clinical changes, we extracted individual eLORETA values and connectivity values from identified brain regions and connectivities using the above-mentioned paired *t*-tests, and we conducted correlation analyses as exploratory analyses. Clinical changes included MADRS reduction volume (post MADRS score − pre MADRS score) and MMSE reduction volume (post MMSE score − pre MMSE score). Statistical analyses were performed using SPSS ver 24.0 (IBM Inc., Armonk, NY, USA). Statistical significance was defined by a *p*-value of <0.05 (two-tailed). Multiple testing corrections were not conducted for the correlation analyses, as these analyses were exploratory.

## Results

### Demographic and Clinical Characteristics

Demographics and clinical characteristics of the participants are summarized in Table 1. Thirteen individuals [five females, mean age: 58.4 (Standard Deviation: 13.6) years old] with unipolar depressive disorder completed this study. After ECT, the total MADRS score was significantly reduced from TP1 to TP2 [TP1: 30.3 (8.6), TP2: 6.5 (6.3), *df* = 12, *t* = 9.04, *p* < 0.001], whereas the total MMSE score did not change [TP1: 27.0 (3.0), TP2: 26.3 (2.9), *df* = 11, *t* = 0.76, *p* = 0.46]. Remission and response rate were 69.2% (9/13) and 92.3% (12/13), respectively. The number of ECT was mean 9.9 (SD: 1.8).

**Table 1:** Clinical characteristics of the participants.

Number of patients	13
Age, years	58.4 (13.6)
Female, *n* (%)	5 (38.5%)
Psychotic features, *n* (%)	5 (38.5%)
Age at onset, years	48.2 (7.7)
Number of depressive episodes	2.5 (1.4)
Duration of current episode, months	10.2 (12.4)
Number of prior antidepressants	4.0 (1.8)
Number of ECT treatments	9.9 (1.8)
Time between the pre-EEG and the first ECT, days	9.1 (7.8)
Time between the last ECT and post-EEG, days	3.1 (1.7)
MADRS total score	
pre-ECT (TP1)	30.3 (8.6)
post-ECT (TP2)	6.5 (6.3)
Clinical Remitters, *n* (%)	9 (69.2%)
Clinical Responders, *n* (%)	12 (92.3%)
MMSE total score	
pre-ECT (TP1)	27.0 (3.0)
post-ECT (TP2)	26.3 (2.9)

*Data are number or mean (standard deviation) unless stated otherwise. Abbreviation: TP1, pre-ECT series (baseline); TP2, post-ECT series (endpoint); ECT, Electroconvulsive therapy; EEG, Electroencephalogram; MADRS, Montgomery Asberg Depression Rating Scale; MMSE, Mini-Mental State Examination*.

### Longitudinal Effects of ECT on Whole Brain CSD

**Figure 1:**
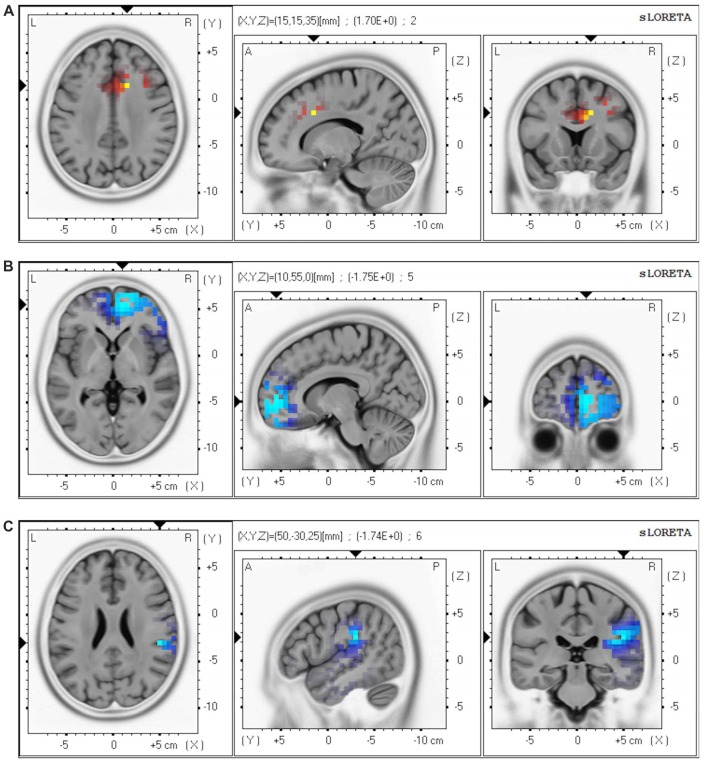
Brain regions showing change in oscillatory patterns after a course of electroconvulsive therapy (ECT). Our analyses revealed the following changes after ECT: **(A)** increased theta (4.0–7.5 Hz) in the anterior cingulate cortex (ACC) and the medial prefrontal cortex (MPFC); **(B)** decreased beta 2 (20.5–30.0 Hz) in the frontal pole (FP); and **(C)** decreased gamma (30.5–45.0 Hz) in the right inferior parietal lobule (IPL). Red regions correspond to significantly increased CSD after ECT, and blue regions correspond to significantly decreased CSD after ECT. Abbreviation: CSD, current source density.

Whole-brain analyses using eLORETA showed the following changes in oscillatory cortical activity patterns after ECT (t_crit_ = 1.52, *p* < 0.05): increased theta (*t* = 1.70) in the ACC and the medial prefrontal cortex (MPFC); decreased beta 2 (*t* = −1.75) in the frontal pole (FP), and decreased gamma (*t* = −1.74) in the right inferior parietal lobule (IPL; Figure 1).

### Longitudinal Effects of ECT on Lagged Phase Synchronization

**Figure 2:**
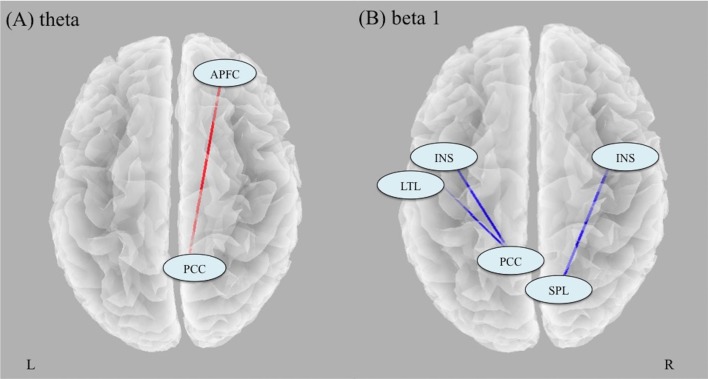
Results of analyses of changes in lagged phase synchronization with ECT. **(A)** There was a significant increase in theta phase synchronization between the right APFC and the right PCC. **(B)** There were significant decreases in the beta 1 phase synchronization between the right INS and the right SPL, between the left PCC and the left INS, and between the left PCC and the left LTL. Abbreviation: APFC, anterior prefrontal cortex; PCC, posterior cingulate cortex; INS, insula; SPL, superior parietal lobule; LTL, lateral temporal lobe.

Analyses of changes in lagged phase synchronization between TP1 and TP2 (t_crit_ = 5.37, *p* < 0.05) revealed that there was a significant increase in theta phase synchronization between the right anterior PFC (APFC) and the right posterior cingulate cortex (PCC; *t* = 5.48). There were significant decreases in the beta 1 phase synchronization between the right insula (INS) and the right superior parietal lobule (SPL; *t* = −5.85), between the left PCC and the left INS (*t* = −6.60), and between the left PCC and the left lateral temporal lobe (LTL; *t* = −5.55; Figure 2).

### Correlation Between Changes in EEG Measurements and Clinical Changes

There were no correlations between changes in CSD in three identified regions (theta CSD in the ACC/MPFC, beta2 CSD in the FP, and gamma CSD in the right IPL) and changes in MADRS or MMSE (Supplementary Table S2). However, connectivity changes between the left PCC and the left INS (*r* = −0.68, *df* = 10, *p* = 0.015) as well as connectivity changes between the left PCC and the LTL (*r* = −0.64, *df* = 10, *p* = 0.024) had negative associations with changes in MMSE.

## Discussion

The current study revealed that ECT increased theta activity in the ACC/MPFC, decreased beta activity in the FP, and decreased gamma activity in the IPL. ECT increased theta phase synchronization between the PCC and the APFC, and decreased beta phase synchronization between the PCC and the temporal regions. We could not find any associations between clinical response and any EEG measurements, but we found a relationship between decreased beta phase synchronization and cognitive change after ECT. This is the first study to show the correlation between ECT-related cognitive change and beta phase synchronization.

### Longitudinal Effect of ECT on Neural Oscillations

We found that ECT increased theta oscillations in the ACC/MPFC and decreased high frequency oscillations in the FP and the right IPL. Since the 1930s, many studies have reported ECT-induced slow wave oscillations in the frontal lobe (Krystal et al., 2000; Farzan et al., 2014), and a recent EEG study reported that ECT decreased high frequency oscillations, especially in patients who responded to ECT (Farzan et al., 2017). Our results are in line with these previous findings, which may support the validity of our findings.

According to fMRI and EEG studies, frontal medial theta activity was negatively correlated with blood oxygen level dependent (BOLD) signals in the default mode network (DMN) regions, namely medial frontal, inferior frontal, precuneus/PCC, inferior parietal, middle temporal cortices, and the cerebellum (Scheeringa et al., 2008). In addition, high-frequency bands, including beta (Laufs et al., 2003) and gamma (Mantini et al., 2007), were positively correlated with DMN BOLD signals. Considering these previous findings, the current results (increased theta in the ACC/MPFC, decreased beta in the MPFC, and decreased gamma in the IPL) may indicate that ECT decreased resting-state electrical activity in nodes of the DMN. A recent meta-analysis of PET studies investigating the effect of treatments for depression (i.e., antidepressant medications and ECT) on brain metabolism revealed that ECT decreased activity in central nodes of the DMN (Chau et al., 2017). Given that electroencephalographic oscillations are a relatively more direct measure of neuronal activity than other modalities (e.g., PET, MRI), the current study may provide additional evidence for the results from previous PET studies.

We could not find any correlations between oscillatory changes in nodes of the DMN regions and ECT and MADRS reduction. One potential interpretation is that an ECT-induced reduction in DMN activity may be just a by-product of electrical stimulation or seizure, and not related to clinical response. Another potential explanation is that a change in DMN activity due to ECT may be related to a change in specific psychiatric symptoms, and not related to a change in the entirety of depressive symptoms (i.e., total HAM-D scores). Since DMN activity is considered to be associated with rumination and autobiographical memory (Zhu et al., 2012), a future study should focus on the relationship between ECT-induced changes in DMN activity and specific symptoms (e.g., rumination) or autobiographical memory, which is known as ECT-related side effects (Semkovska and McLoughlin, 2013).

### Longitudinal Effect of ECT on Lagged Phase Synchronization

ECT-induced EEG slowing suggests that synchronization occurs in the synaptic activity of large neuronal populations, with a reduction in firing rate (Sackeim et al., 1996). Our observed results of ECT-induced increased phase synchronization in theta frequency between the PCC and the APFC (BA9, 10) support this notion. These two regions (PCC and BA9/10) are located in the posterior and anterior central nodes of the DMN, suggesting that ECT may increase phase synchronization within the DMN. Since there were no correlations between changes in theta phase synchronization and those in MADRS and MMSE, the implication of our findings still remains unclear. Therefore, to elucidate the clinical relevance of changes in theta phase synchronization due to ECT, a large sample study that focuses on specific symptoms related to the DMN is needed.

Additionally, the current study revealed that ECT decreased beta phase synchronization between the PCC and the temporal regions. This is in line with a previous EEG study using graph theoretical analysis, which reported that a single session of seizure therapy decreased the phase synchronization in the beta frequency band (Deng et al., 2015). Furthermore, we found a significant correlation between changes in beta synchronization and changes in MMSE scores, which may suggest that depressed patients who present a larger decrease in beta synchronization after ECT show more cognitive decline after ECT. A prior study has also reported that lower beta band synchronization is associated with lower MMSE scores (Stam et al., 2003). Furthermore, the PCC has an important role in autobiographical memory (Leech and Sharp, 2014), which is one of the cognitive functions largely affected by ECT (Semkovska and McLoughlin, 2013). In addition, our finding was restricted to the left hemisphere. The short-term cognitive side effects of ECT change depending on the electrode placement (i.e., bilateral vs. unilateral). Right unilateral electrode placement has been shown to have less cognitive side effects than bilateral electrode placement (Kolshus et al., 2017), and left unilateral electrode placement tended to have more verbal memory impairment than right unilateral electrode placement (Kellner et al., 2017). The interpretation of these results is understandable based on the theory that the left hemisphere is dominant for language and verbal processing for most people. Left-lateralized results in the current study are consistent with this evidence. We used only bilateral electrode placement in the study because our participants were severely depressed patients who needed rapid improvement, but future studies should compare the effects of ECT on neurophysiological and neuropsychological measurements between different electrode placements to test our hypothesis. Taken together, our findings of a decrease in beta synchronization between the left PCC and the left temporal regions may reflect the underlying electrophysiology of ECT-induced cognitive impairments.

## Limitations

The current study should be interpreted with the following limitations. First, the number of participants was limited. A larger study is needed to confirm our preliminary results. Second, all patients continued their psychopharmacological medications, which may affect the EEG oscillatory pattern and phase synchronization. However, a previous study reported that antidepressant medications increased beta band phase synchronization as calculated by LORETA (Olbrich et al., 2014). The effects of ECT on EEG phase synchronization (i.e., ECT decreased beta band phase synchronization) may be stronger than the effects of antidepressant medications. Third, we did not conduct multiple testing corrections for correlation analyses, as the analyses were exploratory. The observed relationship between beta synchronization and cognitive change needs to be replicated. In addition, MMSE includes multiple cognitive domains, so future studies should focus on specific cognitive domains that relate to ECT. Fourth, our sample includes only depressive disorder to avoid heterogeneity, but this also limits the generalizability of our results.

## Conclusion

ECT reduced resting-state EEG oscillatory activity in central nodes of the DMN regions and increased phase synchronization within the DMN. An ECT-induced reduction in beta phase synchronization was associated with the cognitive side effects experienced by patients after a series of ECT.

## Data Availability

The datasets for this study will not be made publicly available because it might be possible if we get consents to provide data from all participants.

## Author Contributions

AT and JH designed the study, recruited the participants, conducted the clinical assessments, and analyzed the data. AT conducted a literature search and wrote the first draft. JH, BY, ST, TK, and MM wrote the final manuscript. All authors contributed to and have approved the final manuscript.

## Conflict of Interest Statement

The authors declare that the research was conducted in the absence of any commercial or financial relationships that could be construed as a potential conflict of interest.

## Abbreviations

CSD: current source density
DMN: default mode network
ECT: electroconvulsive therapy
EEG: electroencephalography
LORETA: low resolution electromagnetic tomography.
