# Supplementary material for: Electroconvulsive Therapy Modulates Resting-State EEG Oscillatory Pattern and Phase Synchronization in Nodes of the Default Mode Network in Patients With Depressive Disorder
Source: Front Hum Neurosci. 2019 Feb 1;13:1. doi: 10.3389/fnhum.2019.00001 (PMC6367251; doi:10.3389/fnhum.2019.00001)
Supplement: Supplementary file 1 [file Data_Sheet_1.docx]

| Supplementary Table 1. Selected 28 ROIs and MNI coordinates | | | | | |
| --- | --- | --- | --- | --- | --- |
| ROI label | | MNI coordinates (x, y, z) | | | |
| Sensorimotor Areas | SMA | left | −40.0 | −25.9 | 50.9 |
|  |  | right | 41.5 | −25.9 | 50.8 |
| Superior Parietal Lobule | SPL | left | −17.1 | −59.8 | 52.1 |
|  |  | right | 15.1 | −59.0 | 51.3 |
| Superior Prefrontal Cortex | SPFC | left | −26.0 | 3.8 | 52.3 |
|  |  | right | 25.0 | 4.7 | 52.4 |
| Anterior Prefrontal Cortex | APFC | left | −25.5 | 34.3 | −15.0 |
|  |  | right | 26.2 | 34.5 | −15.1 |
| Orbitofrontal Cortex | OFC | left | −25.4 | 42.5 | 20.4 |
|  |  | right | 25.1 | 42.6 | 21.4 |
| Lateral Prefrontal Cortex | LPFC | left | −49.5 | 20.4 | 14.5 |
|  |  | right | 50.8 | 21.5 | 14.8 |
| Insula | INS | left | −50.4 | −15.9 | −13.9 |
|  |  | right | 51.0 | −14.7 | −14.2 |
| Lateral Temporal Lobe | LTL | left | −8.3 | 16.2 | 23.1 |
|  |  | right | 6.7 | 14.9 | 22.2 |
| Anterior Cingulate Cortex | ACC | left | −11.1 | −50.9 | 23.0 |
|  |  | right | 8.5 | −49.0 | 25.0 |
| Posterior Cingulate Cortex | PCC | left | −23.6 | −17.7 | −20.8 |
|  |  | right | 23.3 | −17.8 | −20.4 |
| Parahippocampal Gyrus | PHG | left | −48.8 | −47.8 | 34.5 |
|  |  | right | 49.6 | −46.8 | 35.4 |
| Inferior Parietal Lobule | IPL | left | −34.4 | −68.7 | 1.6 |
|  |  | right | 34.5 | −68.7 | 1.2 |
| Fusiform & Lingual Cortex | FLC | left | −16.0 | −85.9 | 0.7 |
|  |  | right | 13.6 | −86.1 | 1.9 |
| Primary Visual Cortex | PVC | left | −38.9 | −7.8 | 8.5 |
|  |  | right | 40.1 | −7.5 | 9.3 |
