# Supplementary material for: Electroconvulsive Therapy Modulates Resting-State EEG Oscillatory Pattern and Phase Synchronization in Nodes of the Default Mode Network in Patients With Depressive Disorder
Source: Front Hum Neurosci. 2019 Feb 1;13:1. doi: 10.3389/fnhum.2019.00001 (PMC6367251; doi:10.3389/fnhum.2019.00001)
Supplement: Supplementary file 2 [file Data_Sheet_2.docx]

| Supplementary Table 2. Correlation between EEG measurements and clinical change. | | | | |
| --- | --- | --- | --- | --- |
|  | MADRS change | | MMSE change | |
|  | r | p | r | p |
| theta CSD in the ACC/MPFC | 0.15 | 0.62 | −0.32 | 0.32 |
| beta CSD in the FP | −0.52 | 0.066 | 0.05 | 0.88 |
| gamma CSD in the right IPL | 0.44 | 0.13 | 0.29 | 0.36 |
| theta connectivity |  |  |  |  |
| right PCC and APFC | −0.41 | 0.16 | −0.31 | 0.33 |
| beta connectivity |  |  |  |  |
| right insula and SPL | −0.009 | 0.98 | −0.11 | 0.73 |
| left PCC and insula | −0.45 | 0.12 | −0.68 | 0.015* |
| left PCC and LTL | −0.46 | 0.11 | −0.64 | 0.024* |

*p <0.05 (uncorrected)

Abbreviation

MADRS: Montgomery Asberg Depression Rating Scale, MMSE: Mini-Mental State Examination, CSD: current source density, ACC: anterior cingulate cortex, MPFC: medial prefrontal cortex, FP: frontal pole, IPL: inferior parietal lobule, PCC: posterior cingulate cortex, APFC: anterior prefrontal cortex, SPL: superior parietal lobule, LTL: lateral temporal lobe
